# The Reconstruction of Magnetic Particle Imaging: Current Approaches Based on the System Matrix

**DOI:** 10.3390/diagnostics11050773

**Published:** 2021-04-26

**Authors:** Xiaojun Chen, Zhenqi Jiang, Xiao Han, Xiaolin Wang, Xiaoying Tang

**Affiliations:** School of Life Science, Beijing Institute of Technology, Beijing 100081, China; 3120185688@bit.edu.cn (X.C.); 3120170646@bit.edu.cn (X.H.); icecreamgao@163.com (X.W.)

**Keywords:** magnetic particle imaging, reconstruction, regularization, system matrix

## Abstract

Magnetic particle imaging (MPI) is a novel non-invasive molecular imaging technology that images the distribution of superparamagnetic iron oxide nanoparticles (SPIONs). It is not affected by imaging depth, with high sensitivity, high resolution, and no radiation. The MPI reconstruction with high precision and high quality is of enormous practical importance, and many studies have been conducted to improve the reconstruction accuracy and quality. MPI reconstruction based on the system matrix (SM) is an important part of MPI reconstruction. In this review, the principle of MPI, current construction methods of SM and the theory of SM-based MPI are discussed. For SM-based approaches, MPI reconstruction mainly has the following problems: the reconstruction problem is an inverse and ill-posed problem, the complex background signals seriously affect the reconstruction results, the field of view cannot cover the entire object, and the available 3D datasets are of relatively large volume. In this review, we compared and grouped different studies on the above issues, including SM-based MPI reconstruction based on the state-of-the-art Tikhonov regularization, SM-based MPI reconstruction based on the improved methods, SM-based MPI reconstruction methods to subtract the background signal, SM-based MPI reconstruction approaches to expand the spatial coverage, and matrix transformations to accelerate SM-based MPI reconstruction. In addition, the current phantoms and performance indicators used for SM-based reconstruction are listed. Finally, certain research suggestions for MPI reconstruction are proposed, expecting that this review will provide a certain reference for researchers in MPI reconstruction and will promote the future applications of MPI in clinical medicine.

## 1. Introduction

**Figure 1:**
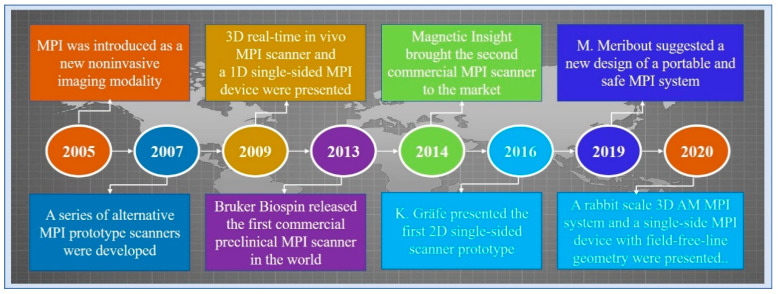
A timeline of MPI history.

Magnetic particle imaging (MPI) is an emerging non-invasive molecular imaging technology that images the concentration distribution of superparamagnetic iron oxide nanoparticles (SPIONs) [1]. In 2005, Gleich and Weizenecker of the Philips Research Laboratory first introduced the imaging principle and application prospects of MPI [2]. In 2007, Conolly and Goodwill developed a series of prototype scanners [3]. In 2009, Weizenecker announced the 3D MPI scanner for the first time [4]. In the same year, Sattel presented a 1D single-sided MPI device [5]. The first 2D prototype of a single-sided scanner was presented by Gräfe [6]. In 2013, the first commercial preclinical MPI scanner was released by Bruker Biospin. In 2014, Magnetic Insight brought the second commercial MPI scanner [1]. Five years later, Meribout and Kalra suggested a new design of a portable and safe MPI system [7]. In 2020, Tuan-Anh Le presented the design of a rabbit scale 3D amplitude modulation MPI system with a bore size of 9 cm [8]. In the same year, Alexey Tonyushkin at the University of Massachusetts Boston proposed a single-side MPI device with field free-line geometry. Figure 1 shows the timeline of MPI history.

MPI directly images the distribution of SPIONs with high temporal-spatial resolution, and the resolution after reconstruction reaches the sub-millimeter level [2]. MPI is a novel imaging modality that outperforms other powerful imaging techniques [9]. The optical imaging modalities, such as the fluorescence imaging, have the problem that the generated signal is surface-weighted. The MPI signal intensity does not decay with the depth and is not limited by the depth of the tissue. The signal strength of positron emission tomography (PET) and single photon emission computed tomography (SPECT) does not decrease with depth. However, the half-life of the radioactive tracers limits their applications in certain research fields, for example, tracking stem cells for weeks or longer [9]. MPI tracks the SPIONs tracer materials with biocompatibility, which can be traced for a long time, can be metabolized, and is easily transformed into humans. For magnetic resonance imaging (MRI), the tracer materials create “black holes” as a negative contrast agent, which may be confused with the air and tissue interfaces or hemorrhages. MPI is able to detect small concentrations of SPIONs in the order of a few ng without tracing any background signal [1]. MPI outperforms X-ray with radiation. Therefore, MPI is a very promising imaging modality, which overcomes the abovementioned shortcomings of traditional imaging techniques. With the continuous improvement of MPI theory and instruments, the application research of MPI in various fields has also made certain progress [10], mainly including multimodal imaging [11], cell tracing [12], drug delivery and monitoring [13], blood pool imaging [14], tumor tracing [15], precision magnetic hyperthermia [16], etc.

**Figure 2:**
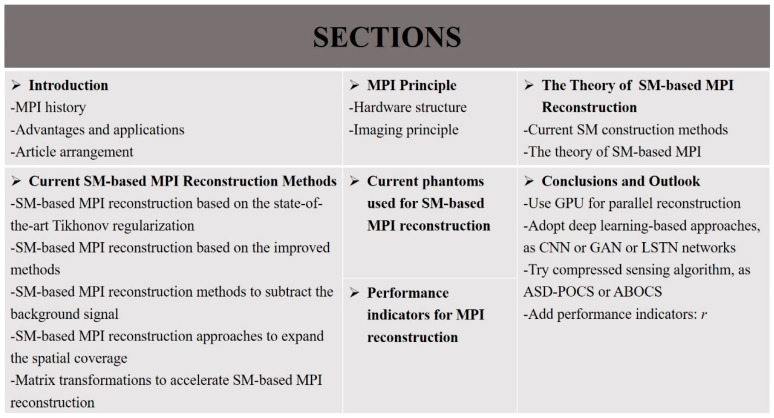
Organization of this work.

The performance of MPI reconstruction is of enormous practical importance to promote its clinical application, and many studies for MPI reconstruction have been explored and conducted to improve the reconstruction performance. There are mainly two methods of MPI reconstruction: the x-space method and SM-based method. MPI reconstruction based on a system matrix (SM) is an important part of MPI reconstruction approaches. In this review, the principle of MPI and the theory of SM-based MPI are discussed. For SM-based approaches, the reconstruction problem usually comes down to solving a series of linear inverse problems, ill-posed in nature by regularization techniques. Different MPI reconstruction methods based on a system matrix are discussed, including an SM-based MPI reconstruction based on the state-of-the-art Tikhonov regularization, SM-based MPI reconstruction based on the improved methods, SM-based MPI reconstruction methods to subtract the background signal, SM-based MPI reconstruction approaches to expand the spatial coverage, and matrix transformations to accelerate SM-based MPI reconstruction. In addition, the current phantoms and performance indicators used for SM-based reconstruction are listed. Finally, certain research suggestions for MPI reconstruction are proposed, expecting that this review will provide a certain reference for researchers in MPI reconstruction and will promote the future applications of MPI in clinical medicine. Figure 2 shows the organization of this work.

## 2. MPI Principle

**Figure 3:**
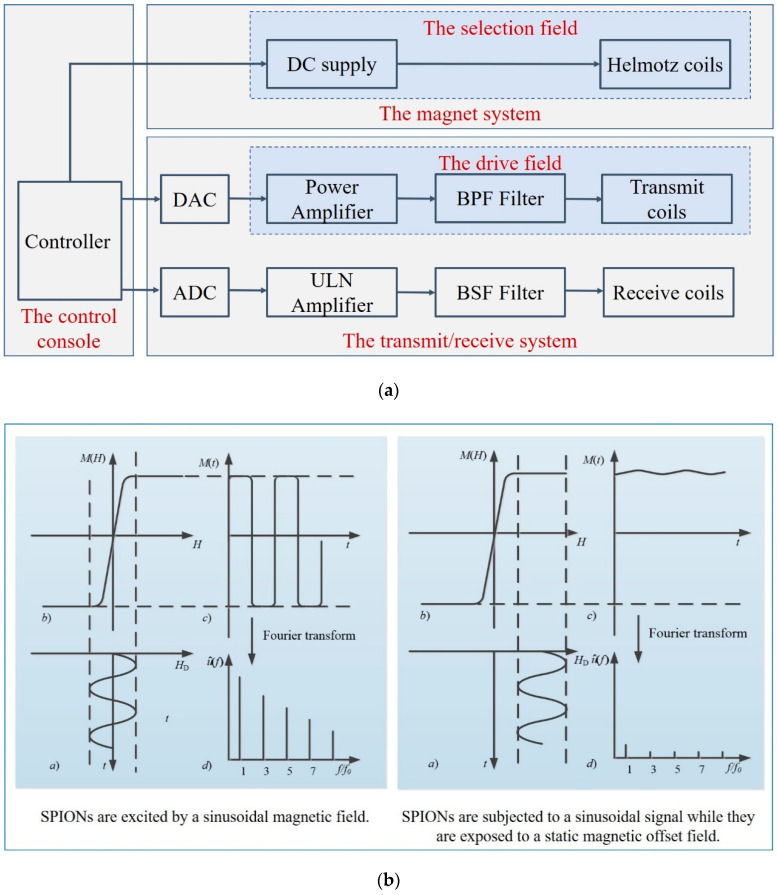
(**a**) Hardware structure of an MPI imaging system; (**b**) the basic principle of MPI.

MPI is based on the nonlinear response of SPIONs to the applied magnetic field. The hardware structure of the MPI system includes three major components: the magnet system, the transmit/receive system, and the control console, as shown in Figure 3a. The control console coordinates the operation of the major subsystems [17]. The magnet system generates the main gradient field by the adjacent parallel Helmotz coils, in which the magnetic field becomes zero, called the field-free region (FFR) [18]. The transmit/receive system generates the drive field and receives the voltage signal. The power amplifier is used to amplify the magnetic field amplitude, and the passive band-pass (BPF) filter is to improve the spectral purity. The band stop filter (BSF) in the receive system rejects the fundamental frequency part of the received signals.

For the imaging process, two different magnetic fields are necessary: the selection field and the drive field [2]. The selection field *H*_s_ is a gradient static magnetic field, and the drive field is a dynamic uniform magnetic field. SPIONs induce a non-linear magnetization when excited by the drive field, and generate a voltage signal in the receive coils. The basic principle of MPI is illustrated in Figure 3b, where (a) shows the drive filed over time *H_D_*(*t*), which is applied on the SPIONs, (b) shows the magnetization curve of SPIONs *M*(*H*), (c) shows the time-varying corresponding magnetization response *M*(*t*), and (d) shows the signal spectrum $\hat{u}(f)$ used for reconstruction. The field-free point (FFP) is produced by two opposite magnetic fields. Only particles inside the FFP or in a direct neighborhood near it do not reach the magnetization saturation state and can be excited, and then generate harmonics of the excitation frequency. In the left, the drive file stimulates the SPIONs near the FFP, whereas, in the right, the SPIONs are in saturation, in which there is almost no change in magnetization over time. Spatial encoding is achieved by moving the FFP throughout the field of view (FOV). The received signal consists of the harmonics of the excitation frequency, which are used to reconstruct the SPION distribution [6,19]. In 2008, Weizenecker first proposed a new scanning method, using a field-free line (FFL) instead of the FFP, which improves the image quality and sensitivity [20]. In 2010, Knopp improved the efficiency of the FFL coil geometry even further [21]. In 2015, Benete presented an electronically rotated FFL scanner [22]. In 2017, Ilbey processed the FFL MPI data via SM or projection-based reconstructions, and compared the relative performance of these two approaches [23]. In 2019, Top analyzed the image quality of different FFL trajectories [18]. In 2020, Top presented the first open-sided MPI system, which can electronically scan the FOV with an FFL [24].

## 3. The Theory of SM-Based MPI

MPI is a new biomedical imaging modality that images the distribution of SPIONs. Some researchers adopt mathematical models to describe the physical process, like the equilibrium model [25] or its variations, the x-space approach [3,26,27,28], Chebyshev polynomials [29], and analytic inversion formulas [30,31]. The state-of-the-art reconstruction techniques adopt the experimentally calibrated forward operators, called the SM-based image reconstruction [32,33]. The SM is the discretized representation of the system function (SF), which describes the mapping of the spatial concentration distribution and the induced voltage signal, including magnetic field characteristics and complex particle characteristics [34]. Therefore, the SM-based MPI reconstruction is more accurate. This section mainly introduces the current SM construction methods and the principle of SM-based MPI.

### 3.1. Current SM Construction Methods

The SM represents the mapping of the spatial concentration distribution and the induced voltage signal. The quality of the SM correlates with the quality of the image reconstruction. The SM can be constructed mainly through two ways, called model-based SM and measurement-based SM.

In 2009, Rahmer studied the structure and properties of the SF used for image reconstruction and proposed to replace the SF based on calibration or measurement with an SF based on Chebyshev polynomial modeling [35]. In 2010, Knopp first proposed a model-based SM acquisition method using the normalized root mean square error (NRMSE) to verify the similarity between the model-based system matrix and the measurement-based system matrix [36]. The average NRMSE of all frequency components is around 10%. In 2017, Marz performed mathematical modeling on the reconstruction of two-dimensional and three-dimensional MPI and obtained a reconstruction formula different from one-dimensional [30]. In the same year, Knopp described the basic principles of MPI imaging, including signal generation, spatial coding, and signal detection [37]. In 2018, Kluth supplemented the model and studied linear and nonlinear problems in concentration reconstruction [25]. Compared with the measurement-based SM, the model-based SM is with application flexibility to hypothetical particle models, etc., avoiding the repeated calibration process that is necessary for building the measurement-based SM and greatly reduces the construction time of SM, but it is difficult to ensure the construction accuracy.

The measurement-based approach is a common way to obtain the SM [6,38,39]. To measure the SM of a MPI scanner, a cube-shaped calibration sample is used that is similar to electrical capacitance tomography (ECT) [40]. The voxel-sized calibration sample is moved vertically and horizontally over FOV, therefore, the SF can be obtained by measuring the system response of a delta probe at every voxel. In addition, the measurement-based system matrix needs to be corrected for the background signal, and the measurement is usually interrupted every 20 s to detect the background signal [41]. To enhance the signal-to-noise ratio (SNR) of the calibration measurements and reduce the calibration time, Ilbey presented the coded calibration scene (CCS) framework, where SPION samples are place inside the FOV in a random or pseudo-random fashion [42].

The measurement-based SM is preferred to avoid deviations linked to the model-based reconstruction approaches [22,38,43]. This method is the most well-known and the easiest solution especially for inhomogeneous magnetic fields. In addition, the interaction between particles and the dynamic characteristics of magnetic nanomaterials can be better described in the measurement-based SM. Therefore, the measurement-based SM can describe the mapping between the SPION concentration distribution and the receiving end signal more accurately.

### 3.2. The Theory of SM-Based MPI

MPI images the spatial concentration distribution of SPIONs, and the reconstruction of MPI is done to solve the spatial concentration distribution of SPIONs. The image reconstruction based on SM is performed in frequency space [6,44]. A discrete Fourier transformation is performed on the recorded voltage signal *u*(*t*) directly after the data acquisition, expressed as ${\hat{u}}_{k}$. In the space Ω with magnetic nanoparticles, the relation of the particle concentration *c*(***r***), the coefficients ${\hat{u}}_{k}$, and the SF ${\hat{s}}_{k}(r)$ is described as [36]:

(1) $${\hat{u}}_{k}=\int_{\Omega }{\hat{s}}_{k}(r)c(r)dr$$

When dispersing the space Ω into *N* points $r_{n}\in \Omega$,$n\in I_{N}$, *I_N_* represents a set length *N* and *I_N_* = 0,1…*N*-1, then, the discretization of Equation (1) is expressed as [29]:

(2) $${\hat{u}}_{k}=\sum_{n\in I_{n}}{\hat{s}}_{k,n}c_{n}$$

Here, ${\hat{s}}_{k,n}:=\omega_{n}{\hat{s}}_{k}(r_{n})$, $c_{n}:=c(r_{n})$ represents the value of the SF and the concentration at the spatial position ***r***_n_, respectively, and $\omega_{n}=d_{x}d_{y}d_{z}$ is the orthogonal weight.

In a matrix form, the inverse problem between the induced voltage signal, the system matrix, and the particle concentration can be expressed as [6,38,45]:

*u* = *Sc* (3)

with system matrix $S:={\left({\hat{s}}_{k,n}\right)}_{k\in I_{K},n\in I_{N}}\in {\mathbb{C}}^{K\times N}$, the desired particle concentration $c:={\left(c_{n}\right)}_{n\in I_{N}}\in {\mathbb{R}}_{+}^{N}$, and the measured induced voltage sensed by receiving coils $u:={\left({\hat{u}}_{k}\right)}_{k\in I_{K}}\in {\mathbb{C}}^{K}$, where *K* is the number of frequencies used for MPI reconstruction. The columns of ***S*** represent the spatial positions of the system acquisition, and therefore cover the FOV. The rows of ***S*** represent the frequency components at all spatial positions.

The matrix compression technology or threshold setting method are usually used to construct the sparse or truncate system matrix. The relation between the truncated system matrix $\hat{S}$, the truncated measurement $\hat{u}$, and the SPION concentration distribution ***c*** can be expressed as [6]:

(4) $$\hat{u}=\hat{S}c$$

## 4. Current SM-Based MPI Reconstruction Methods

There have been some studies dedicated to improving the spatial resolution and image quality of MPI, including optimizing the particle size distribution of nanoparticles [46,47], simulating different scanning trajectories [18,48], building scanners with stronger gradient fields [49], adopting the new calibration protocol [50], and improving the reconstruction approaches [45], etc. Different SM-based reconstruction methods have been investigated in different studies. For SM-based approaches, MPI reconstruction mainly has the following problems: the reconstruction problem is an inverse and ill-posed problem [51], the complex background signals seriously affect the reconstruction results, the FOV could not cover the entire object, and the available 3D datasets are of a relatively large volume. In this review, we compared and grouped different studies on the above issues, including SM-based MPI reconstruction based on the state-of-the-art Tikhonov regularization, SM-based MPI reconstruction based on the improved methods, SM-based MPI reconstruction methods to subtract the background signal, SM-based MPI reconstruction approaches to expand the spatial coverage, and matrix transformations to accelerate SM-based MPI reconstruction.

### 4.1. SM-Based MPI Reconstruction Based on the State-of-the-Art Tikhonov Regularization

The SM-based MPI reconstruction is an inverse and ill-posed problem. Therefore, regularization is needed to solve the concentration distribution and realize image reconstruction. The Tikhonov regularization is the most popular approach, and is widely used in MPI reconstruction, which can be performed by solving a series of linear equations [6]. Thus, it is fast and simple to implement. The MPI reconstruction based on the Tikhonov method can be solved via direct or iterative methods, such as singular value decomposition (SVD) [51], the conjugate gradient (CG) method [52], and the Kaczmarz method [7]. Besides avoiding the high initial cost for decomposition based on direct solvers, a further advantage of an iterative algorithm like the Kaczmarz method is that it allows the incorporation of physical constraints, such as a real non-negative particle concentration *c* in the iteration procedure. Usually, the number of Kaczmarz iterations is chosen between 1 and 10 [53]. It converges rapidly due to the orthogonality of the system matrix, and results in a reasonable image quality [54]. The best ratio between the reconstruction quality and reconstruction time were achieved after several iteration steps, with five iteration steps in [54]. Therefore, the state-of-the-art idea for SM-based reconstruction is the standard Tikhonov regularization solved by the Kaczmarz iteration. Schmiester [29] and Grafe [6] adopted the Tikhonov regularization for SM-based reconstruction and solved the ill-posed problem with the Kaczmarz iteration. Knopp also proposed to combine this popular idea with preconditioning (row normalization) and row exclusion for improving the reconstruction quality [55].

The desired particle concentration can be extracted from the weighted least squares approach, combined with an iterative solver [6], expressed as:

(5) $${\left\|\hat{S}c-\hat{u}\right\|}_{W}^{2}={\left\|W^{1/2}(\hat{S}c-\hat{u})\right\|}_{2}^{2}\underset{\to }{c}min$$

*W* is the weighting matrix used to normalize the entries of the SM and suppress the components with bad SNR. In addition, λ is the regularization parameter. The identity matrix *I* and the conjugate transpose of the truncated system matrix ${\hat{S}}^{*}$ are introduced as follows:

(6) $$({\hat{S}}^{*}W\hat{S}+\lambda I)c={\hat{S}}^{*}W\hat{u}$$

The reconstruction results of [6] show that almost all of the bright blocks can be reconstructed based on the Tikhonov method. However, in the reconstructed images, more artifacts appear, accompanied by the desired bright blocks. The artifacts have to be eliminated to improve the reconstruction accuracy and quality. As mentioned above, the Tikhonov regularization is particularly simple to implement. On the downside, the limited noise suppression capability directly hampers some biomedical applications, such as bolus tracking and vessel visualization. For facilitating fast and accurate MPI reconstruction, T. Kluth and B. Jin proposed two algorithmic tricks from the inverse perspective [56]: one is a whitening procedure to incorporate the noise statistics for improving the reconstruction accuracy, and the other is the randomized SVD for accelerating the Kaczmarz iteration. They are easy to implement and straightforward to incorporate in the existing reconstruction techniques. In the same year, they also reviewed several issues in MPI reconstruction, i.e., the choice of data fidelity, choosing a suitable regularization parameter, and accelerating the Kaczmarz iteration via randomized SVD. The experiments on a publicly available dataset show significant potentials.

### 4.2. SM-Based MPI Reconstruction Based on the Improved Methods

For the Tikhonov regularization, the spatial neighborhood structures of the underlying image are not reflected in the prior. This drawback can be overcome by applying the *l*_1_ norm prior. In particular, total variation (TV) [57] and the least absolute shrinkage and selection operator (LASSO) regularization [45,58] have been used for various imaging modalities, like MRI [59], PET [60], and X-ray CT [61].

The LASSO algorithm was proposed by Tibshirani in 1996. This method overcomes the shortcomings of the Tikhonov regularization through the *l*_1_ norm prior, and the mathematical expression is:

(7) $${\begin{array}{cc} arg & \min\beta \left\|c\right\| \end{array}}_{1}+\frac{1}{2}{\left\|\hat{S}c-\hat{u}\right\|}_{2}^{2}$$

Currently, more advanced regularization techniques, such as fused LASSO penalty [45], approximation error modeling [62], and deep image prior (DIP) [63], have been proposed and empirically evaluated for MPI reconstruction. These improved variational regularization methods integrate model errors into the reconstruction process, which are more compatible with the statistical characteristics of the image and are significantly better than the Tikhonov method in MPI reconstruction. Martin Storath developed an efficient edge-preserving and denoising reconstruction method for MPI [45]. As the regularization technique, they proposed to adopt the LASSO model. Furthermore, they proposed to use the TV prior for taking the neighborhood structure into account. In addition, they imposed non-negativity constraints for the particle density. Therefore, the following advanced model for MPI regularization is proposed:

(8) $$\arg\underset{u\geq 0}{\min}\alpha TV(c)+\beta {\left\|u\right\|}_{1}+\frac{1}{2}{\left\|\hat{S}c-\hat{u}\right\|}_{2}^{2}$$

where TV(***c***) is the total variation and ${\left\|c\right\|}_{1}$ denotes the *l*_1_ norm. The regularization parameters *α*, *β* > 0 control the relative regularizing weight. Without the non-negativity, (8) is known as the fused LASSO. Henceforth, they defined the model (8) as non-negative fused LASSO. The system matrix is small when in one dimension, and the Chambolle–Pock algorithm can be used to solve the above model. However, the system matrix is huge when in two dimensions or three dimensions, and the Chambolle–Pock algorithm needs to evaluate the near-end mapping of the data item in each iteration, which is time-complex. Furthermore, the gradient intensity of the magnetic gradient field applied in high dimensions is not necessarily the same in all directions. They also designed a discretization suitable for the acquisition geometry, and developed a customized solver of the forward–backward scheme.

The reconstruction results based on the non-negative fused LASSO were compared with the Tikhonov regularization for three phantoms with one percent Gaussian noise: a simulated stenosis, overlapping ellipses, and a vascular tree. For three phantoms, the non-negative fused LASSO model provides a better quality than the Tikhonov method with respect to the visual inspection, NRMSE, and SSIM. They also corrupted the data with 5, 10, and 15 percent Gaussian noise to compare the robustness of the proposed method. The results showed that the reconstructed images retain the homogeneity and sharp boundaries, even in the case of 15 percent noise.

Chae investigated the MPI reconstruction based on a neural network, and confirmed that a multi-layer with one hidden layer could improve the reconstruction performance [64]. DIP networks have been recently introduced in deep learning for applications in image processing [65]. For ill-posed Equation (4), the task of DIP is to train a network with parameters by minimizing the simple loss function. Training with a single data point is the most striking property, which separates DIP from other neural network concepts. Network architectures can be interpreted as a minimization algorithm that solves a regularized inverse problem. Dittmer studied the so-called deep image prior techniques of ill-posed inverse problems and first reported the experimental results for applying DIP to inverse problems [66].

### 4.3. SM-Based MPI Reconstruction Methods to Subtract the Background Signal

Consistent with other imaging methods, the imaging quality of MPI is affected by background signals, which may originate from the thermal process of the scanner electronics, such as the amplifier, filter, coils, or the contamination of ferromagnetic tracers [67,68]. The standard measurement method of the background is measuring the SM and system response separately without the tracer, and then subtracting the background signal from the system response measured with the tracer. In the MPI imaging process, the background signal is not constant [41,69], and the complex background signal will seriously affect the reconstruction results. Therefore, the time interval between the successive background measurements needs to be short enough, such as a background interpolation of every 20 s [41].

MPI reconstruction is performed in the frequency domain. The frequency components of the truncated induced voltage signal $\hat{u}$ include three parts: the real particle response ${\hat{u}}^{true}$, the background signal ***b***, and the Gaussian noise ***n***.

The three are independent of each other, expressed as:

(9) $$\hat{u}={\hat{u}}^{true}+b+n$$

The noise signal ***n*** can be averaged through multiple measurements to reduce the impact on image reconstruction, and the background signal ***b*** is usually measured without tracers. Theoretically, the real particle signal can be obtained by averaging the noise signal and subtracting the background signal after multiple measurements. However, the background signal ***b*** changes with time. This method is only suitable for a sufficiently small time interval between the background measurement and sample measurement. Straub and Schulz performed MPI reconstruction by adopting the traditional linear interpolation method with the Kaczmarz algorithm, and proved that there is a significant difference between the two reconstructed images at a time interval of 55 min [39].

**Figure 4:**
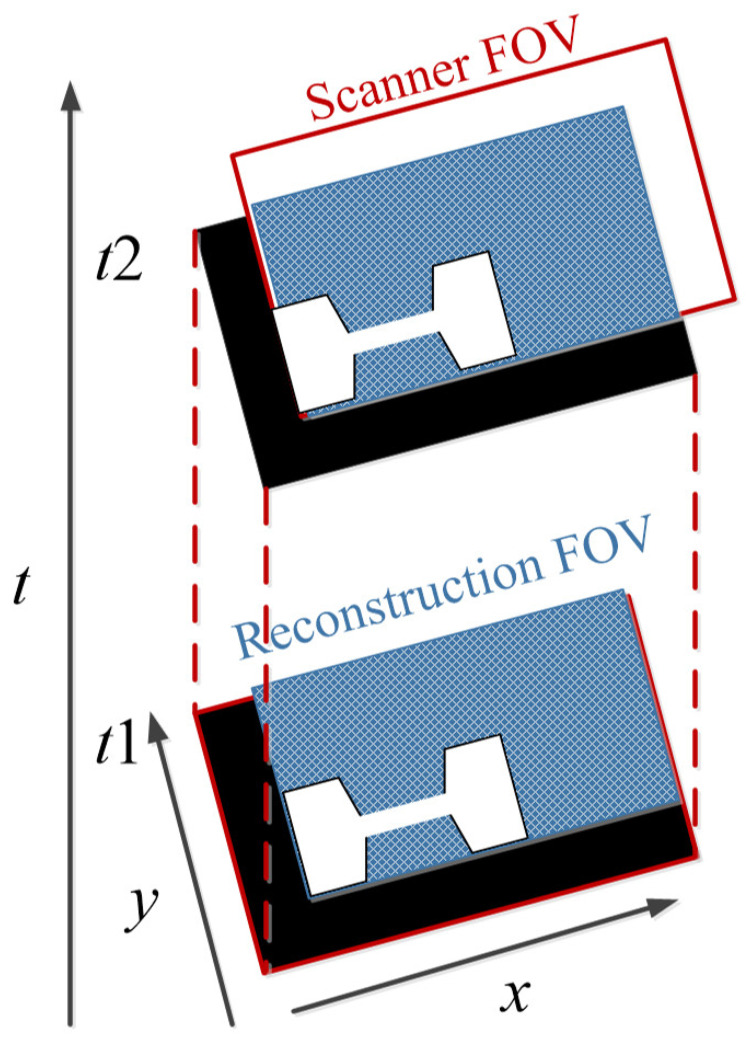
The schematic diagram of 2D joint image reconstruction.

To avoid the interruption of the image acquisition process, Straub and Schulz studied the joint MPI reconstruction method of the tracer distribution and background [39]. Based on the high time resolution of MPI, they assumed that the tracer distribution and background of the two consecutive images are almost the same, and the FOV shifts between the consecutive images are introduced to realize the separation of the background signal and the tracer signal. The scanner FOV can be moved between two consecutive images by superimposing a uniform magnetic field (such as the focus field). The two-dimensional joint image reconstruction method is shown in Figure 4, and the time series of image acquisition are displayed vertically. The FOVs (marked in red) of the scanner at *t*_1_ and *t*_2_ move one voxel in the spatial direction, and the FOV used for the reconstruction is the superposition of two FOVs (marked in blue).

The concentration distribution is solved with the mixed signal ${\hat{u}}_{i}$ by the least square form, expressed as:

(10) $$\sum_{i=1}^{2}{\left\|W(Sc(r_{i})-u_{i}+b)\right\|}_{2}^{2}+\alpha {\left\|c\right\|}_{2}^{2}+\beta {\left\|b-b_{init}\right\|}_{2}^{2}\to \min$$

where $\beta {\left\|b-b_{init}\right\|}_{2}^{2}$ (β≥0) represents the prior of the background spectrum and ***b****_init_* is the background measured before.

The iterative algorithm of L-BFGS-B (imitated memory Broyden–Fletcher–Goldfarb–Shanno) was adopted to solve the least square problem, and ***c*** was non-negatively constrained. The stopping criterion is that the maximum norm of the gradient is less than 10^−1^. To verify the advantages of the proposed joint method, Straub and Schulz conducted the simulation study compared to the Kaczmarz method. The signal-to-background ratio (SBR) was used as the evaluation index to evaluate the degree of background suppression. The MPI reconstruction based on Kaczmarz has lots of artifacts and high noise with low contrast. The image reconstruction based on the proposed joint method was with high contrast. The significant difference of the reconstruction results is also reflected in the SBR. The SBR of MPI reconstruction based on the joint method is 38.4, and the SBR of MPI reconstruction based on Kaczmarz is 11.8. On the other hand, the MPI reconstruction based on the joint method for 25 × 25 voxel takes 40 s, while the Kaczmarz method takes 0.1 s, which is an obvious deficiency of this method.

### 4.4. SM-Based MPI Reconstruction Approaches to Expand the Spatial Coverage

**Figure 5:**
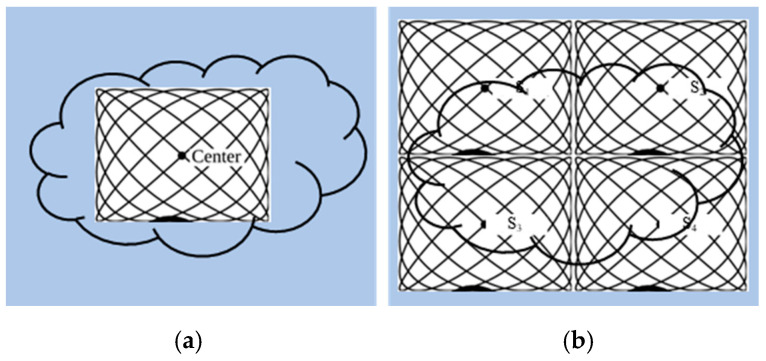
(**a**) The phantom cannot be covered by a single patch; (**b**) the phantom can be covered by multiple patches.

The size of the FOV depends on the excitation amplitude and gradient strength. The commonly applied amplitude of the drive field is 10 mTμ_0_^−1^, and the gradient strength of the selection field varies from 0.5 Tm^−1^μ_0_^−1^ to 7 Tm^−1^μ_0_^−1^ [70]. This leads to a spatial coverage of about 3 mm to 40 mm. However, it is impossible to apply a higher amplitude due to peripheral nerve stimulation (PNS) or tissue heating [71,72]. Therefore, if the volume of the object exceeds 40 mm, then the FOV will not cover the entire object. In order to expand the spatial coverage, the so-called focus fields can be applied. By sampling larger scan volumes either patch by patch or in a continuous manner, it is possible to avoid the limitation of the small drive field amplitude. Therefore, several FOVs (called patches) at different locations are measured to cover the larger areas (Figure 5).

In this method, multiple patches are merged into a joint approach for reconstruction. The data of multiple patches are combined based on the coupling between patches, and the concentration distribution is solved by the joint method [69]. In addition, overlapping patches can be applied to reduce the truncation artifacts [38]. For large objects, the number of necessary patches is high. In general, the size of the linear system increases exponentially with the number of patches, making it more demanding for the main memory of the reconstruction computer. Therefore, the disadvantage of this method is that the main memory of the reconstruction computer is more demanding. P. Szwargulski et al. developed a reconstruction algorithm for MPI multi-patches, applying the sparsity of the joint SM [38]. The proposed reconstruction algorithm combined with a highly efficient implicit matrix format were applied to two different 3D phantoms, which were acquired by a preclinical MPI Scanner (Bruker, Ettlingen, Germany). Both of the phantoms were designed with a 3D CAD software and measured by 15 patches to cover the entire FOV. The regularized Tikhonov algorithm was used to perform MPI reconstruction. Using this approach combined with an implicit matrix format, the computational effort was reduced to a linear dependence on the number of used patches. The reconstruction of 15 patches was realized within 22 s.

However, in this joint reconstruction method, parts of the entire FOV are measured twice, which decreases the scanning efficiency. Furthermore, the joint reconstruction approach assumes that the target concentration remains unchanged between scanning the different patches. For example, the movement of a patient may violate this assumption. In the case of measuring several patches, the truncation artifacts are reduced by applying overlapping patches. However, only inner border artifacts can be processed. In practice, SPIONs outside the FOV can also be excited, leading to the domain truncation artifacts. Brandt and Seppänen proposed a computational technique for reducing the truncation artifacts [62]. They cast the MPI reconstruction in the Bayesian framework and applied the Bayesian approximation error modeling (AEM) method. For the numerical experiments, they used three different phantoms. The typical border artifacts were suppressed, and the computational effort was lower.

Another way to bypass the limitation of the small strength of the applied magnetic fields is external object movements. In 2018, Szwargulski investigated this approach [73], where an object is moved through the scanner bore one step at a time, and the data could be acquired continuously from the static FOV of the MPI scanner. The experiments of 3D phantom and in vivo data showed that the data could be jointly reconstructed after reordering the data.

### 4.5. Matrix Transformations to Accelerate SM-Based MPI Reconstruction

The SM-based MPI reconstruction is done to invert the concentration distribution of SPIONs using the induced voltage signal. It is worth noting that the available 3D datasets are of a relatively large volume. Many important efforts have been made to accelerate MPI reconstruction based on matrix compression [29,74,75,76].

Grafe adopted the signal-to-noise ratio (SNR) threshold method for component reduction of SM [6]. To ensure better reconstruction quality and accuracy and shorter reconstruction time, only frequency components of SNR are used for reconstruction. Thus, for the reconstruction process, a truncated SM $\hat{S}$ and a truncated measurement $\hat{u}$ are used. To calculate the SNR, two system matrices have been measured, an empty one without SPIONs (SE) and one with SPIONs (SP) [6].

The energy of each frequency component *f* at each spatial position *r* is given by:

(11) $$\omega_{f,r}=\left\|SP_{f,r}-SE_{f,r}\right\|$$

Using the standard deviation $\sigma_{f}$ of the SE, the SNR is calculated:

(12) $$SNR=\frac{\omega_{f,r}}{\sigma_{f}}$$

The SNR threshold is chosen so that the reconstruction results in visually appropriate images. Studies have shown that by using a Philips MPI scanner when the threshold is set to 10, the removed frequency components can reduce the noise of the reconstructed image by about 90% [77].

For a multidimensional imaging system, the structure of SM is not fully explored due to the complexity of the matrix structure caused by the sampling trajectory. Weber and Knopp proved the row symmetry of 2D SM, similar to the tensor product of Chebyshev polynomials, and mathematically proved the feasibility of 2D matrix compression [78]. They also combined the symmetry with compressed sensing to reduce calibration scans by approximately three times [41]. Since the SM is ill-conditioned, the regularization term does not change the computational complexity of the reconstruction problem [76]. The dimensionality of the forward map can be reduced by using a row selection technique based on the SNR type quality measure. Knopp and Hofmann proposed an online and adaptive reconstruction framework, which allowed direct visualization of the SPIONS distribution, with a latency of about 2 s. In order to optimize the signal quality and adapt the throughput of the reconstruction process, block averaging is performed without skipping data.

The other approach for SM compression is to use the sparse approximations of the linear forward graph, which is achieved by applying discrete orthonormal transformations. Lampe et al. proposed a new reconstruction method to accelerate the MPI reconstruction based on effective matrix compression and an iterative algorithm [74]. The matrix compression was based on the orthogonal transform, such as the 3D discrete cosine transform and the 3D discrete Chebyshev transform. The useful information of SM could be extracted through the threshold processing, and then the conjugate gradient least squares (CGLS) and least squares QR decomposition (LSQR) sparse approximation were adopted to accelerate the iteration. They also studied the effects of matrix compression on memory requirements, the computational complexity, and the image quality. For 4D MPI, the real-time reconstruction with almost no quality loss was realized, and the reconstruction time was shortened by 50–500 times. Schmiester et al. also introduced the matrix compression technology and applied the Chebyshev transform to transform the SM into the sparse domain. They also studied the direct reconstruction based on the Chebyshev transform, and verified that the Chebyshev transform is more suitable for MPI matrix compression than the cosine transform, and can provide better reconstruction quality [29].

## 5. Current Phantoms Used for SM-Based MPI Reconstruction

Many studies based on different phantoms have been conducted to improve reconstruction accuracy and quality. Table 1 shows the different phantoms used for MPI reconstruction.

## 6. Performance Indicators for MPI Reconstruction

To evaluate the reconstruction quality, different performance indicators have been used, as shown in Table 2, where *I*(*i*,*j*) and *U*(*i*,*j*) represent the intensity of the reconstructed image and original image, respectively.

## 7. Conclusions and Outlook

MPI is an emerging molecular imaging modality that images the distribution of SPIONs with high sensitivity and resolution. The plentiful applications of MPI for diagnosis and treatment have been explored. MPI reconstruction is of enormous practical importance. SM-based MPI reconstruction is an important part of MPI reconstruction. Presently, there are mainly two ways to construct the SM: measurement-based SM and model-based SM. Measurement-based SM is preferred to avoid deviations linked to model-based reconstruction, especially for inhomogeneous magnetic fields. In this review, different reconstruction methods based on the system matrix have been reviewed, including SM-based MPI reconstruction based on the state-of-the-art Tikhonov regularization, SM-based MPI reconstruction based on the improved methods, SM-based MPI reconstruction methods to subtract the background signal, SM-based MPI reconstruction approaches to expand the spatial coverage, and matrix transformations to accelerate SM-based MPI reconstruction. Furthermore, the current phantoms and performance indicators used for SM-based reconstruction are listed, including the absolute mean error/NRMSE/SSIM/the reconstruction time *t*.

It is worth noting that it is very necessary to accelerate MPI reconstruction with improved quality. We suggest improving the existing reconstruction algorithm in parallel, by using GPU for parallel reconstruction, increasing the reconstruction speed, and realizing real-time reconstruction. In addition, reconstruction accuracy is as important as the speed, and MPI reconstruction based on recent methods is with a lot of background artifacts. The deep learning-based approaches are very active in the biomedical image reconstruction area. The CNN, GAN, or LSTN networks are very widely used in this line of research [80,81]. We believe that those models could be used in future experiments for result improvements. The compressed sensing (CS) algorithm is to attribute the reconstruction to the sparse optimization problem under the regularization theory. The CS reconstruction algorithms for CT, such as adaptive steepest descent projection onto convex sets (ASD-POCS) and accelerated barrier optimization compressed sensing (ABOCS), etc., can be applied to MPI reconstruction. Regarding the evaluation of MPI reconstruction results, we suggest that the performance indicators of traditional MRI/CT reconstruction can be added, such as the Pearson correlation coefficient (*r*) and the peak signal-to-noise ratio (PSNR). The *r* describes the strength of the linear correlation between two variables, which can be used to evaluate the similarity between the reconstructed image and original image. The PSNR is the most common and widely used objective measure for image processing. The higher the PSNR, the better the reconstruction results.

Based on the imaging principle of MPI, researchers have proposed a variety of data processing methods and image reconstruction algorithms, and have verified the advantages of these algorithms through practical testing and simulation analysis. Compared with mature medical images, such as MRI/CT/PET, the MPI reconstruction method is still under exploration, and there are still many problems to be further studied. Presently, the accuracy and quality of MPI reconstruction still need to be improved, and the reconstruction time still needs to be reduced for real-time imaging. Reducing the artifacts of MPI reconstruction, shortening the time of MPI reconstruction, and improving the accuracy and quality of MPI reconstruction have great practical significance for promoting the real-time imaging and clinical application of MPI. As more scientists enter the field of MPI reconstruction research, MPI is believed to soon experience a period of rapid development. With the further development of MPI devices, tracers, and reconstruction methods, the clinical application of MPI will be expanded. We expect that this review may provide a certain reference for researchers in MPI reconstruction and promote the future applications of MPI in clinical medicine.

## Figures and Tables

**Table 1:** Different phantoms used for MPI reconstruction.

Year	Journal	Phantoms	Description
2016	IEEE Transactions on Medical Imaging	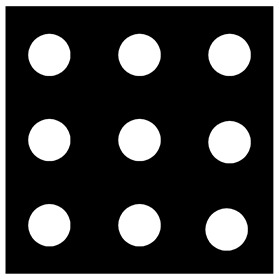	The size of the cube-shaped calibration sample size is 2 × 2 × 2 mm^3^. The calibration sample is moved in vertical and horizontal steps of 2 mm over the 30 × 30 mm^2^ FOV [6].
2017	IEEE Transactions on Medical Imaging	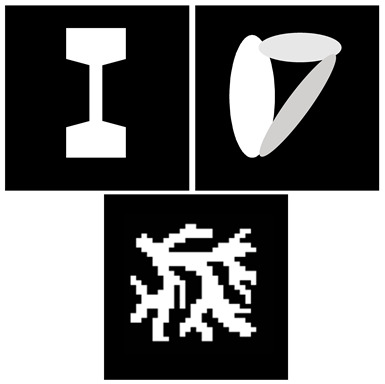	Three different phantoms with one percent Gaussian noise are used to evaluate the reconstruction quality: a simulated stenosis, overlapping ellipses, and a vascular tree [45].
2018	Journal of Mathematical Imaging and Vision	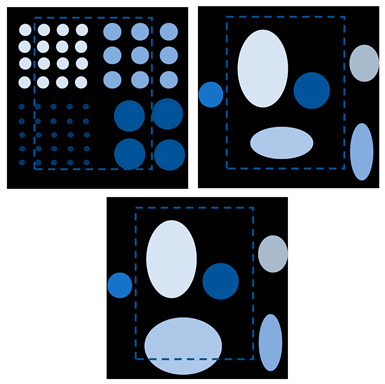	The first is a typical resolution phantom with round objects of different size and concentration. The second includes three ellipses with different size and concentration. The third simulates a situation where objects cannot be covered by a single FOV [62].
2019	IEEE Transactions on Medical Imaging	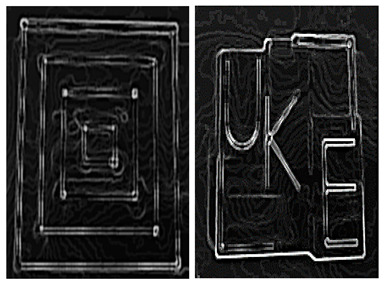	The first is the filled 3D-printed model, which consists of four rectangles with different sizes. The second is the UKE phantom. The letters of the phantom are located in different planes in the y direction [38].
2019	Physics in Medicine & Biology	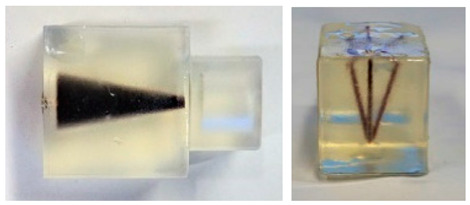	These two phantoms are from the open MPI datasets (www.tuhh.de/ibi/research/open-mpi-data.html (accessed on 7 October 2020)). The first is a cone and the second consists of five tubes with a common origin on one side of the phantom [56].
2019	Measurement	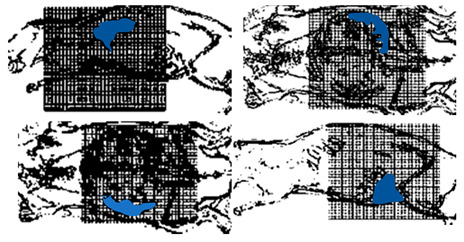	Real images are used to study MPI reconstruction, which represent the different mouse organs: the lungs, left kidney, right kidney, and reproductive system [7].

**Table 2:** Different performance indicators used for evaluating the reconstruction quality.

Journal	Indicator	Equation and Description
Measurement	the absolute mean error	$Error=\left[\frac{1}{X*Z}\sum_{i=1}^{X}\sum_{j=1}^{Z}\left|I(x,j)-U(x,j)\right|\right]*100\left[\%\right]$ The absolute mean error is a quantitative evaluation [7]. It considers eventual image transforms, such as rotation, translation, and zoom transform [45]. Therefore, it is fairer.
IEEE Transactions on Medical Imaging	NRMSE	$NRMSE=\sqrt{\frac{\sum_{i=1}^{X}\sum_{j=1}^{Z}{\left[I(x,j)-U(x,j)\right]}^{2}}{\sum_{i=1}^{X}\sum_{j=1}^{Z}{\left[I(x,j)\right]}^{2}}}$ The NRMSE is an objective evaluation index of image quality based on pixel error. It reflects the degree of difference between the reconstructed image and the original image [45]. The smaller the NRMSE, the better the reconstruction quality.
IEEE Transactions on Medical Imaging	SSIM	*SSIM* (*I*, *U*) = *L(I*,*U*) × *C*(*I*,*U*) × *S*(*I*,*U*) $L\left(I,U\right)=\frac{2u_{I}u_{Y}+C_{1}}{u_{I}^{2}+u_{U}^{2}+C_{1}}$ $C\left(I,U\right)=\frac{2\sigma_{I}\sigma_{U}+C_{2}}{\sigma_{I}^{2}+\sigma_{U}^{2}+C_{2}}$ $S\left(I,U\right)=\frac{\sigma_{IU}+C_{3}}{\sigma_{I}\sigma_{U}+C_{3}}$ where *u_I_* and *u_U_* represent the mean values of images *I* and *U*, respectively. *σ_I_* and *σ_I_* represent the standard deviations of image *I* and *U*. *σ_IU_* represents the image *I* and *U* covariance. *C*1, *C*2, and *C*3 are constants to prevent the denominator from being 0 and maintain stability. SSIM takes the similarity of local structures into account [45], therefore, it is more suitable for perceiving visual quality. The SSIM is limited by 1, and a higher SSIM means a better reconstruction result [79].
Physics in Medicine & Biology	The reconstruction time *t*	The reconstruction time represents the solution time of equation $\hat{u}=\hat{S}c$, where $\hat{S}$ and $\hat{u}$ represent the truncated system matrix and truncated measurement, respectively. The shorter *t* is, the better the reconstruction performance. The reconstruction time is always the mean of the execution time for 100 times [56].
